# The diagnostic ability of SPECT/CT fusion imaging for gastrointestinal bleeding: a retrospective study

**DOI:** 10.1186/s12876-018-0915-7

**Published:** 2018-12-10

**Authors:** Yoichi Otomi, Hideki Otsuka, Kaori Terazawa, Moriaki Yamanaka, Yuki Obama, Maki Arase, Maki Otomo, Saho Irahara, Michiko Kubo, Naoto Uyama, Takashi Abe, Masafumi Harada

**Affiliations:** 1 Department of Radiology, Tokushima University Hospital, 2-50-1 Kuramoto-cho, Tokushima, Tokushima 770-8503 Japan; 2 Department of Medical Imaging/Nuclear Medicine, Institute of Biomedical Sciences, Tokushima University Graduate School, 2-50-1 Kuramoto-cho, Tokushima, 770-8503 Japan

**Keywords:** Gastrointestinal bleeding, Gastrointestinal bleeding scintigraphy, Tc-99 m HSA-DTPA, SPECT/CT

## Abstract

**Background:**

Blood loss from the gastrointestinal tract can be an acute and life-threatening event. For the treatment of gastrointestinal bleeding, it is important to accurately detect gastrointestinal bleeding and to localize the sites of bleeding. The purpose of this study was to retrospectively assess the capabilities of SPECT/CT in the diagnosis of gastrointestinal bleeding by a comparison with planar imaging alone as well as planar and SPECT.

**Methods:**

We conducted a retrospective analysis of 20 patients (21 examinations) who underwent gastrointestinal bleeding scintigraphy in the past 7 years and in whom the bleeding site was identified by endoscopy or capsule endoscopy, or in whom no evidence of gastrointestinal bleeding was identified during the clinical course. Five patients (5 examinations) were diagnosed by planar imaging (planar group). Eight patients (9 examinations) were diagnosed by planar imaging and SPECT (planar + SPECT group). Seven patients (7 examinations) were diagnosed by planar imaging and SPECT/CT (planar + SPECT/CT group). We calculated the diagnostic ability of each method in detecting the presence of bleeding, as well as the ability of each method to identify the sites of bleeding. The sensitivity, specificity, and accuracy of the methods were compared.

**Results:**

The diagnostic ability of the three imaging methods in detecting the presence of gastrointestinal bleeding was as follows. Planar imaging showed 100% sensitivity (3/3), 100% specificity (2/2), and 100% accuracy (5/5). Planar + SPECT imaging showed 85.7% sensitivity (6/7), 100% specificity (2/2), and 88.9% accuracy (8/9). Planar + SPECT/CT imaging showed 100% sensitivity (6/6), 100% specificity (1/1), and 100% accuracy (7/7). The diagnostic ability of the three modalities in detecting the site of bleeding was as follows: planar, 33.3% (1/3); planar + SPECT, 71.4% (5/7); and planar + SPECT/CT, 100% (6/6).

**Conclusions:**

All 3 imaging methods showed good accuracy in detecting the presence of gastrointestinal bleeding. The addition of SPECT or SPECT/CT made the anatomical position of the uptake clear and contributed to the localization of the site of gastrointestinal bleeding. Planar + SPECT/CT imaging therefore showed the highest diagnostic ability for detecting the site of gastrointestinal bleeding.

## Background

Gastrointestinal bleeding is a problem commonly encountered in the clinical setting. In most cases, bleeding will stop spontaneously without any intervention; however, blood loss from the gastrointestinal tract can represent an acute and life-threatening event. Obscure gastrointestinal bleeding (OGIB) is defined as bleeding of unknown origin that persists or recurs after an initial negative endoscopic evaluation, including upper and lower endoscopy. OGIB can be classified as overt or occult OGIB. Overt OGIB refers to recurrent or persistent visible bleeding (hematochezia, melena or hematemesis), and occult OGIB is defined as recurrent or persistent iron deficiency and/or positive fecal occult blood [1, 2].

For the treatment of gastrointestinal bleeding, it is necessary to localize the bleeding site. Although methods of identifying the bleeding site vary, endoscopy is usually performed to localize the site. Other methods include angiography, computed tomography (CT), capsule endoscopy and scintigraphy. Angiography has the advantage of therapeutic intervention through transcatheter embolization, but cannot detect bleeding at a rate of < 1.0 ml/min [3, 4]. Intermittent bleeding is difficult to detect by angiography [5]. The accuracy of the angiography in detecting the site of bleeding is reported to range from 43 to 87% [6, 7]. Contrast enhanced CT is used to detect gastrointestinal bleeding. The extravasation of contrast agent into the bowel lumen generally represents bleeding. If bleeding is not active or is intermittent at the time of imaging, the usefulness of this method is limited [8]. In a study using a swine model, a colonic bleeding rate of 0.3 ml/min was detectable on contrast enhanced CT [9]. Capsule endoscopy revolutionized the examination of the small intestine, and has been established as one of the procedures used to diagnose small intestine bleeding [10, 11]; the diagnostic yield is 40–60% [12, 13].

Bleeding scintigraphy is a nuclear medicine imaging examination that does not require bowel preparation and enables the detection of gastrointestinal bleeding with a bleeding rate as low as 0.1–0.5 ml/min [1, 12]. In this examination, a radioisotope is injected intravenously. This method is safe and minimally invasive and allows for continuous monitoring over several hours, which is a major advantage over other diagnostic methods. In addition to Tc-99 m-labeled red blood cells (Tc-99 m RBCs), Tc-99 m human serum albumin-diethylenetriaminepentaacetic acid (Tc-99 m HSA-DTPA) has been widely used in gastrointestinal bleeding scintigraphy [14–16]. Scintigraphy with Tc-99 m HSA-DTPA is a blood pooling method similar to scintigraphy with Tc-99 m RBCs. Tc-99 m HSA-DTPA is stable in blood and does not generate free Tc-99 m.

The addition of single-photon emission computed tomography/computed tomography (SPECT/CT) to planar imaging on gastrointestinal bleeding scintigraphy can help to identify the site of gastrointestinal bleeding [14, 17]. To date, no studies have compared the diagnostic ability of planar + SPECT/CT imaging and planar + SPECT imaging. Thus, in the present study, we calculated the diagnostic ability of each method for detecting the presence of bleeding as well as the ability of each method to identify the sites of bleeding. The sensitivity, specificity, and accuracy of the methods were compared.

## Methods

### Patients

Twenty patients who underwent a total of 21 scintigraphic examinations because of gastrointestinal bleeding over the 9-year period from March 2007 to September 2015 were retrospectively included in the study. The bleeding site was identified by endoscopy (*n* = 11), capsule endoscopy (*n* = 2) or contrast-enhanced CT (n = 2), or else no evidence of gastrointestinal bleeding was identified during the clinical course (*n* = 5). The study group comprised 9 female patients and 11 males (mean age ± standard deviation [SD] = 65.7 ± 18.1 years; range 16–85 years). Among the 20 patients of the study group, 45% (*n* = 9) had overt OGIB, and 45% (n = 9) patients had occult OGIB. The remaining 10% (n = 2) were not classified with regard to OGIB because they did not undergo endoscopic examinations. In five patients, only planar imaging was performed. These patients were therefore defined as the planar group. In eight patients, planar and SPECT imaging were performed. One of these patients was examined twice using this method. These patients were therefore defined as the planar + SPECT group. In seven patients, planar and SPECT/CT imaging were performed. These patients were therefore defined as the planar + SPECT/CT group. We retrospectively reviewed the gastrointestinal bleeding scintigraphy of the 20 patients (21 scintigraphy examinations) to investigate the diagnostic ability of the three imaging modalities. This retrospective study was approved by the institutional review board, which waived the requirement for written informed consent.

### Scanning and data acquisition for gastrointestinal bleeding scintigraphy

All patients were injected with Tc-99 m HSA-DTPA (Nihon Medi-Physics Co. Ltd., Tokyo, Japan) through the peripheral vein. Planar images of the anterior and posterior sides of the abdomen and pelvis were acquired. Initially, 64 frames were obtained consecutively for blood perfusion phase dynamic imaging at a rate of 2 s/frame (matrix size, 128 × 128 pixels). After blood perfusion phase dynamic imaging, static imaging was performed at 10, 20, 30, 40, 50, 60 min, and 3, 6, 24 h (matrix size, 256 × 256 pixels). SPECT data were obtained when an abnormal uptake of RI was suspected based on the planar imaging findings. When an abnormal uptake of RI was not detected on planar imaging, SPECT data were obtained at 6 h and 24 h. SPECT data were acquired for the region of interest (matrix size, 128 × 128 pixels, 6° angle steps, 20 s/frame). The acquisition parameters for CT were as follows: 130 keV, pitch 1.0, rotation time 0.6 s, and slice thickness 5.0 mm.

### The planar group and the planar + SPECT group

A dual-head gamma camera (E.CAM, Toshiba Medical Systems Corporation, Otawara, Japan) with a low-energy high-resolution (LEHR) collimator was used in the planar group and the planar + SPECT group.

### Planar + SPECT/CT group

A SPECT/CT system (Symbia T16, Siemens Medical Solutions, Erlangen, Germany) with a LEHR collimator was used in the planar + SPECT/CT group.

### Evaluation of gastrointestinal bleeding scintigraphy

The 21 scintigraphy examinations of the 20 patients were interpreted by two board-certificated nuclear medicine physicians. Two physicians specializing in nuclear medicine performed the interpretation and all final judgments were made by consensus. We evaluated the ability of detecting the presence of bleeding and the ability of identifying the bleeding sites.

### Statistical methods

The quantitative variables were expressed as mean ± standard deviation (SD) with the ranges. The normality of distribution of the variables was tested by the Levene test. A one-way analysis of variance (normal distribution) or the Kruskal-Wallis test (non-normal distribution) was applied to evaluate differences in parameters. The SPSS Statistics software program (version 24, IBM, Chicago, IL, USA) was used to perform the statistical analyses. *P* values of < 0.05 were considered to indicate statistical significance.

## Results

The clinical characteristics of the patients in the present study are shown in Table 1. The bleeding site was identified in 16/21 (76.2%) examinations. The bleeding site was identified in 3/5 (60%) examinations in the planar group, 7/9 (77.8%) examinations in the planar + SPECT group, and 6/7 (85.7%) examinations in the planar + SPECT/CT group. No bleeding sites were identified in the other 5 examinations. The sites and causes of bleeding are shown in Table 2. The bleeding sites in the 16 examinations included the small intestine (*n* = 9), ascending colon (*n* = 2), stomach (n = 2), rectum/anus (*n* = 1) and extra-gastrointestinal tube (n = 2). The causes of bleeding in the 16 examinations included ulcer (*n* = 5; small intestine [*n* = 4], colon [n = 1]), vascular ectasia (*n* = 3), metastatic carcinoma (n = 2), rupture of varices (n = 2), diverticulum (n = 1), fistula (n = 1) and extra-gastrointestinal lesions (n = 2; subcutaneous hematoma [n = 1], rupture of aneurysm [n = 1]).

**Table 1:** The clinical characteristics of the study group (*n* = 21)

	Planar (*n* = 5)	Planar + SPECT (*n* = 9)	Planar + SPECT/CT (*n* = 7)	*p* value
Age	59.4 ± 23.6	63.6 ± 20.9	71.6 ± 8.8	0.512
Gender (male)	3 (60%)	5 (56%)	3 (43%)	0.838
Blood test				
Hgb (g/dl)	7.8 ± 1.5	8.7 ± 2.0	9.2 ± 1.7	0.429
Hct (%)	23.5 ± 4.3	27.3 ± 6.4	28.8 ± 4.5	0.268
Plt (× 10^3^/μl)	286 ± 73	211 ± 127	290 ± 103	0.426
BUN (mg/dl)	40.4 ± 44.7	16.9 ± 6.3	18.0 ± 4.3	0.494
Cre (mg/dl)	1.65 ± 2.30	0.83 ± 0.24	1.05 ± 0.57	0.896
Drug use				
NSAIDs	1	2	4	
Steroid	3	3	1	
Antiplatelet drug	0	2	2	
Anticoagulant	1	3	0	
Past medical history				
Renal failure	1	0	3	
Heart failure	1	2	0	
Liver cirrhosis	1	0	2	
Malignancy	2	5	4	
Post gastrointestinal op. status	2	3	4	
Inflammatory bowel disease	1	1	0	

*Hgb* Hemoglobin, *Hct* Hematocrit, *Plt* Platelets, *BUN* Blood urea nitrogen, *Cre* Serum creatinine, *NSAIDs* Nonsteroidal anti-inflammatory drugs

**Table 2:** The sites and causes of bleeding

Sites		Causes	
Small intestine	9	Ulcer	5
Ascending colon	2	Vascular ectasia	3
Stomach	2	Metastatic carcinoma	2
Rectum/Anus	1	Rupture of varices	2
		Diverticulum	1
		Fistula	1
Extra-gastrointestinal tube	2	Subcutaneous hematoma	1
		Rupture of aneurysm	1

The diagnostic ability of the three methods in detecting the presence of gastrointestinal bleeding was as follows. The planar group showed 100% sensitivity (3/3), 100% specificity (2/2), a PPV of 100% (3/3), an NPV of 100% (2/2), and 100% accuracy (5/5). The planar + SPECT group showed 85.7% sensitivity (6/7), 100% specificity (2/2), a PPV of 100% (6/6), an NPV of 66.7% (2/3), and 88.9% accuracy (8/9). The planar + SPECT/CT group showed 100% sensitivity (6/6), 100% specificity (1/1), a PPV of 100% (6/6), an NPV of 100% (1/1), and 100% accuracy (7/7) (Table 3).

**Table 3:** The diagnostic ability in detecting the presence of bleeding

	Sensitivity	Specificity	PPV	NPV	Accuracy
Planar group (*n* = 5)	100% (3/3)	100% (2/2)	100% (3/3)	100% (2/2)	100% (5/5)
Planar + SPECT group (*n* = 9)	85.7% (6/7)	100% (2/2)	100% (6/6)	66.7% (2/3)	88.9% (8/9)
Planar + SPECT/CT group (*n* = 7)	100% (6/6)	100% (1/1)	100% (6/6)	100% (1/1)	100% (7/7)

*PPV* Positive predictive value, *NPV* Negative predictive value

**Fig. 1:**
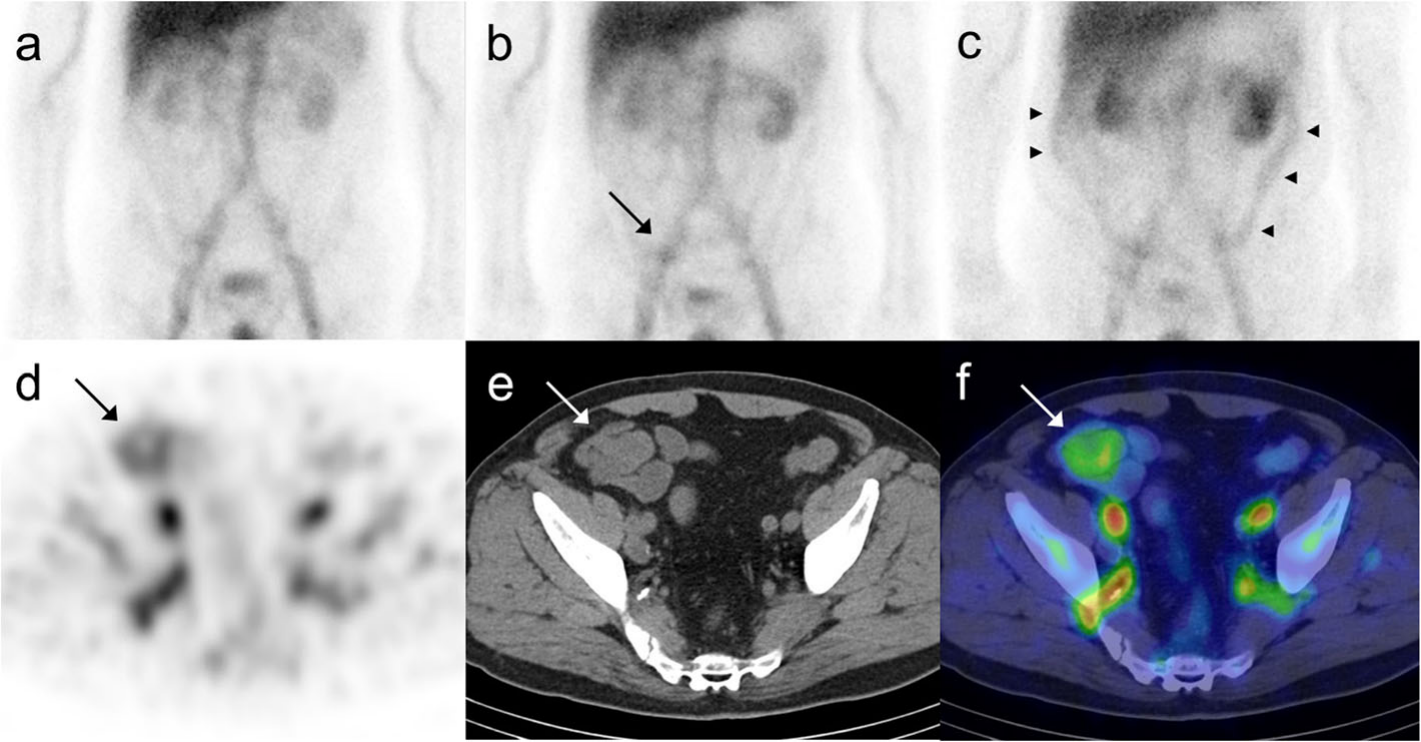
Planar + SPECT/CT of the representative patient where the site of bleeding could be detected. Planar images (**a** 3 h after radioisotope injection, **b** 6 h after, **c** 24 h after) and SPECT/CT images (**d** SPECT, **e** CT, **f** SPECT/CT, 6 h after radioisotope injection). No abnormal uptake was noted in planar images at 3 h after radioisotope injection (**a**). A spotty uptake was seen in the right lower abdomen in planar images at 6 h after the injection (arrow) (**b**). In the planar images at 24 h after the injection, a diffuse uptake was seen in the ascending and descending colon (arrowheads) (**c**). In SPECT/CT images at 6 h after the injection, the spotty uptake matched the distant jejunum (**d**, **e** and **f**) (arrows). It was revealed to be small intestinal metastasis of colon cancer

The diagnostic ability of planar, planar + SPECT and planar + SPECT/CT in detecting the site of bleeding was 33.3% (1/3), 71.4% (5/7) and 100% (6/6), respectively (Table 4). Figure 1 show a representative patient in whom both the presence and site of bleeding was detected by planar + SPECT/CT.

**Table 4:** The diagnostic ability in detecting the site of bleeding

	No. of correctly identified bleeding sites	No. of incorrectly identified bleeding sites	Accuracy
Planar group (*n* = 3)	1	2	33.3% (1/3)
Planar + SPECT group (*n* = 7)	5	2	71.4% (5/7)
Planar + SPECT/CT group (*n* = 6)	6	0	100% (6/6)

In the planar images, we found an abnormally increased uptake that indicated bleeding on early images (< 60 min after RI injection) in 11/16 (68.8%) examinations, and on delayed images (> 180 min after RI injection) in 4/16 (25.0%) examinations. Another 1 examination was a false-negative (Table 5).

**Table 5:** The positive uptake in the gastrointestinal bleeding-positive examinations

	Planar Early images (< 60 min after RI injection)	Planar Delayed images (> 180 min after RI injection)	SPECT
Planar group (*n* = 3)	3	2	–
Planar + SPECT group (n = 7)	5	6	6
Planar + SPECT/CT group (*n* = 6)	3	6	6

*RI* Radioisotope

## Discussion

The most common bleeding site was the small intestine and most common cause of bleeding was ulcer. There were two examinations in which the site of bleeding was located outside the gastrointestinal tube. One involved a lumbar subcutaneous huge hematoma and the other involved the intraperitoneal rupture of a left gastric artery aneurysm. Gastrointestinal bleeding scintigraphy images of the 2 examinations showed an abnormally increased uptake and positivity for bleeding; however, we misjudged the bleeding site in the case involving the intraperitoneal rupture of a left gastric artery aneurysm, which was in the planar group. Gastrointestinal bleeding scintigraphy could detect bleeding outside of the gastrointestinal tube.

We would thus like to emphasize that an abnormally increased uptake, which indicated bleeding, was detected in the early images of most examinations. Schillaci et al. reported that an abnormal uptake indicating bleeding was detected in early images (< 90 min after RI injection) in 18 of 20 patients (90%) [17]. Because an abnormal uptake indicating bleeding was detected earlier in most cases, special care should be taken in the interpretation of early images.

All 3 groups (planar group, planar + SPECT group, planar + SPECT/CT group) showed good accuracy in detecting the presence of gastrointestinal bleeding. Kotani et al. reported that the accuracy of planar imaging in detecting bleeding was high as 83% [14]. Although each of the imaging methods was good for detecting the presence of gastrointestinal bleeding, not all these methods were good for detecting the site of bleeding. In the present study, the planar group showed low diagnostic ability (33.3%) in detecting the site of bleeding. The diagnostic ability of the planar + SPECT group (71.4%) was superior to that of the planar group. Olds et al. reported that the accuracy of planar images on Tc-99 m-RBC scintigraphy in detecting the site of bleeding was 48% [18], while Schillaci et al. reported that the accuracy of planar images on Tc-99 m-RBC scintigraphy in detecting the site of bleeding was 42.8% [17].

There are some case reports that additional SPECT images of gastrointestinal bleeding scintigraphy were useful for localizing the site of bleeding [16, 19, 20]; however, few studies have focused on the comparison of SPECT to planar gastrointestinal bleeding scintigraphy. In our study, planar + SPECT was superior to planar imaging in detecting the site of bleeding. Because additional SPECT images provide more information than anatomic cross-sectional imaging modalities such as CT, the additional performance of SPECT can help to identify the site of gastrointestinal bleeding.

In the previous articles that compared SPECT/CT to planar gastrointestinal bleeding scintigraphy, planar + SPECT/CT was superior to planar imaging in detecting the site of bleeding. The accuracy of planar + SPECT/CT in the localization of gastrointestinal bleeding site was reported to be 78%, while that of planar imaging was 50% [14]. No articles have compared SPECT to SPECT/CT. Our study was the first to compare the diagnostic ability of SPECT/CT with that of SPECT. In evaluating gastrointestinal bleeding scintigraphy in daily medical practice, we interpret both planar image and SPECT or SPECT/CT image. Planar image can demonstrate entire abdomen and pelvis, and SPECT or SPECT/CT can show anatomical location of bleeding site in more detail. Therefore, we compared planar + SPECT/CT with planar + SPECT.

Although the detectability of the bleeding site in the planar group was low (33.3%), the detectability in the planar + SPECT and planar + SPECT/CT groups was high (71.4 and 100%, respectively). With the addition of SPECT or SPECT/CT, the anatomical position of the uptake becomes clear. In particular, planar + SPECT/CT imaging showed the highest diagnostic ability of the three methods in detecting the site of gastrointestinal bleeding.

This study has a few limitations. First, this study was performed in a single institution, and relatively few cases were evaluated. Given that the limited data currently available might have resulted in some inaccuracies in our findings, further studies in a larger series of cases is required to more fully elucidate the clinical advantages of SPECT/CT for the diagnosis of gastrointestinal bleeding. Second, the series of gastrointestinal bleeding disease includes deflection because this is a retrospective study. Third, the endoscopic exams performed varied among cases. A fourth limitation relates to the absence of a comparison with CT angiography, which has demonstrated high degrees of accuracy for the diagnosis of active gastrointestinal bleeding, and with CT-enteroclysis, which has demonstrated high degrees of accuracy for the diagnosis of obscure gastrointestinal bleeding [2, 21–23]. However, despite these limitations, the present study is the first time compared planar + SPECT/CT of gastrointestinal bleeding scintigraphy with planar + SPECT in detecting the bleeding site. Our study was the first that compare the diagnostic ability of SPECT/CT with SPECT.

## Conclusion

In the three groups (planar group, planar + SPECT group, planar + SPECT/CT group) examinations showed high degrees of accuracy in detecting the presence of gastrointestinal bleeding. Moreover, our results indicate that planar + SPECT/CT imaging has higher diagnostic ability for detecting the site of gastrointestinal bleeding than the other two methods. Therefore, we conclude that gastrointestinal bleeding scintigraphy using plane + SPECT/CT images might be more useful for determining the treatment plan of gastrointestinal bleeding than the other two methods.

## Abbreviations

CT: Computed tomography
LEHR: Low-energy high-resolution
OGIB: Obscure gastrointestinal bleeding
SPECT: Single photon emission computed tomography
Tc-99 m HSA-DTPA: Tc-99 m human serum albumin-diethylenetriaminepentaacetic acid
Tc-99 m RBCs: Tc-99 m-labeled red blood cells
